# Burden of Diabetes Mellitus in the Medically Underserved Rio Grande Valley

**DOI:** 10.7759/cureus.70088

**Published:** 2024-09-24

**Authors:** John M Gaddis, Elias Arellano, Kassandra Pulido, Tyler Torres, Dominic Chau-Zanetti, Natasha Quailes, Andres R Suarez Parraga

**Affiliations:** 1 Orthopedic Surgery, University of Texas Rio Grande Valley School of Medicine, Edinburg, USA; 2 Internal Medicine, University of Texas Rio Grande Valley School of Medicine, Edinburg, USA; 3 General Surgery, University of Texas Rio Grande Valley School of Medicine, Edinburg, USA

**Keywords:** diabetes, medically underserved, obesity, prevalence, public health, rio grande valley

## Abstract

Introduction

Diabetes mellitus (DM) encompasses metabolic disorders characterized by elevated blood sugar. This study aimed to evaluate the prevalence and associated metrics of DM in the Rio Grande Valley (RGV), a low-income and medically underserved region in the United States, and compare these metrics to the national averages from 2012 to 2022.

Methods

A retrospective cross-sectional analysis was conducted using publicly accessible data from the Centers for Medicare and Medicaid Services (CMS). Metrics analyzed included DM prevalence, average principal cost, rates of emergency department visits, hospitalizations, screenings, and prevalence of obesity. Data from the RGV counties were compared to national averages using Mann-Whitney U tests, with a p-value of <0.05 considered significant.

Results

From 2012 to 2022, DM affected patients in the RGV (43.95%) at significantly higher rates than the national average (26.73%) (p < 0.001). Obesity prevalence in the RGV was at higher rates than the national average (24.41% vs. 15.55%, p < 0.01). The screening rates of DM exceeded the national average (10.64% vs. 5.09%, p < 0.001). The average principal cost for patients in the RGV ($1,920.45) to treat DM was significantly greater than the national average principal cost ($859.64) (p < 0.001). The RGV also reported higher rates of ED visits (16.82 vs. 8.82 per 1,000 beneficiaries, p < 0.001) and hospitalizations (7.75 vs. 3.82 per 1,000 beneficiaries, p < 0.001).

Conclusion

The RGV exhibits significantly higher rates of DM and DM-associated metrics compared to the national averages, highlighting substantial public health disparities.

## Introduction

Diabetes mellitus (DM) refers to a spectrum of metabolic disorders arising from insufficient insulin secretion, diminished insulin sensitivity, or a combination of both, leading to elevated blood sugar levels [1]. Most cases of DM are categorized into two main groups: type 1 or type 2 DM. Type 1 DM results from an autoimmune response that causes a decrease in insulin secretion, while type 2 DM stems from insulin resistance with eventual impaired insulin function [1]. Chronic DM can lead to serious complications, including coronary artery disease, peripheral artery disease, carotid artery disease, retinopathy, neuropathy, and nephropathy [1, 2].

The prevalence of DM worldwide has steadily risen in the adult population from 4.7% in 1980 to 8.5% in 2014, affecting over 422 million people at that time [3]. Furthermore, in 2020, it was estimated that 37.3 million Americans, or 11.1% of the population, were living with a diagnosis of type 2 DM in the United States [4]. The increase in obesity rates and reduced physical activity levels have been linked to the surge in prevalence of DM [3, 5]. Previous studies have also identified an association between the prevalence of DM and income levels, with DM disproportionately affecting lower-income areas compared to those of middle and high income [3, 6].

In the United States, the Rio Grande Valley (RGV) is a notable example of both a low-income and medically underserved region [7]. Approximately 30% of its residents live below the poverty line, while 40% lack health insurance coverage [7, 8]. Furthermore, this region faces shortages of healthcare professionals, health services, and health centers [7, 9]. The RGV, characterized by an 85%-90% Hispanic demographic, comprises Hidalgo, Cameron, Starr, and Willacy counties [7, 8]. Previous studies have shown an increased prevalence of various disorders in the RGV when compared to the national average, including cervical cancer, Kaposi sarcoma, osteoporosis, and Alzheimer's disease, among others [7-11]. Yet, to our knowledge, there are no prior studies comparing the rates of DM and related metrics across the four counties of the RGV to the national average.

Therefore, this study aimed to compare the prevalence of DM and various associated metrics across the four counties of the RGV with national averages from 2012 to 2022. These metrics can potentially offer a more complete representation of both DM and the overall health landscape in the region. We hypothesize that the prevalence of DM, along with all DM-related metrics in the RGV, will significantly exceed the national average.

## Materials and methods

Institutional Review Board approval was not required for this retrospective cross-sectional study, as the data used were publicly accessible. The ‘Mapping Medicare Disparities by Population’ tool from the Centers for Medicare and Medicaid Services (CMS) (Data.CMS.gov) was employed to analyze Medicare beneficiary data pertaining to DM-related metrics from 2012 to 2022 [12]. A comparative analysis of the prevalence, average principal cost, rates of ED visits, hospitalizations, screenings, as well as the prevalence of obesity, was conducted between the four counties of the RGV and national averages. Covariates were selected for analysis as potential predictors of the state of DM within the region. 

The CMS defines the metrics used in the analysis in ‘The Mapping Medicare Disparities Tool, Technical Documentation’ [12]. Prevalence rates were determined using specific diagnosis codes from the International Classification of Diseases - Ninth Revision (1CD-9) and International Statistical Classification of Diseases and Related Health Problems: Tenth Revision (ICD-10) [13,14] in Medicare beneficiaries’ claims, while the average principal cost is the yearly mean of all expenses across various types of claims with a primary diagnosis associated with that particular condition [12]. The rate of ED visits is determined as the number of ED visits for specific revenue center codes in a given year per 1,000 beneficiaries, using both inpatient and outpatient data regardless of whether the patient was subsequently hospitalized [12]. The hospitalization rate was calculated by the number of inpatient hospital discharges per 1,000 beneficiaries annually based on principal diagnosis codes found in Medicare administrative claims [12]. Rates for diabetes screening were determined by the frequency of Medicare beneficiaries using preventative services included under the Healthcare Common Procedure Coding System codes: 82947, 82950, and 82951 [12].

This dataset used the Medicare Fee-For-Service population from 2012 to 2022. Prevalence, average principal cost, ED visit rates, hospitalization, and preventative services were measures used in the study. The adjustment was unsmoothed age-standardized, analyzing both the base measure and difference from the national average and a domain of primary chronic conditions. Conditions utilized included diabetes and diabetes screening. All patients eligible under Medicare were included in the study, with no exclusions based on sex, age, or ethnicity.

Data analysis was conducted using Anaconda Navigator (Anaconda Software Distribution. Computer software. Vers. 2.5.0. Anaconda, Sept. 2023, Web. <https://anaconda.com>.), Jupyter Notebook version 6.4.5 (Jupyter Team, https://jupyter.org/), and Python version 3.9 (Python Software Foundation, Fredericksburg, VA). Dataframes were created with the respective data for the variables which included prevalence, average principal cost, rates of ED visits, hospitalizations, and diabetes screenings. 

Preliminary statistics were utilized to ensure that the data met the assumptions of normality. A Shapiro-Wilks test was utilized via the function scipy.stats.shapiro to ensure the data fit a normal distribution. Additionally, the functions matplotlib.pyplot as plt, scipy.stats import, and scipy.stats.mannwhitneyu were utilized to create plots and perform the Mann Whitney-U tests for the data obtained from the ‘Mapping Medicare Disparities by Population’ tool [12]. A Mann-Whitney-U test was utilized to compare the prevalence, average principal cost, rates of ED visits, hospitalizations, and diabetes screening means between the RGV and national average. A p-value of <0.05 was considered significant for all statistical analyses.

## Results

Diabetes mellitus and obesity prevalence

**Figure 1:**
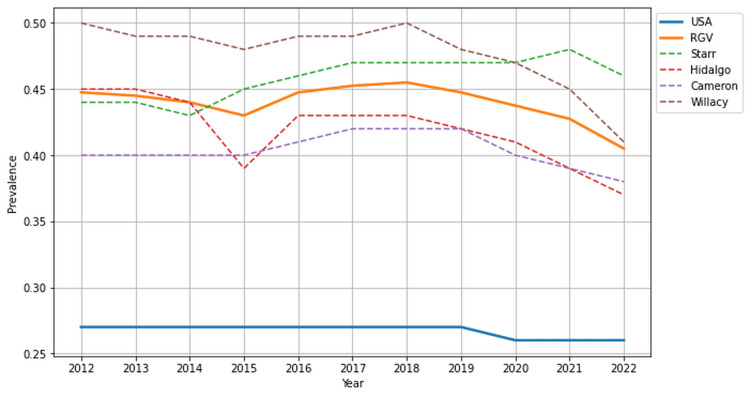
Prevalence of diabetes mellitus in the USA and Rio Grande Valley (RGV) from 2012 to 2022

**Figure 2:**
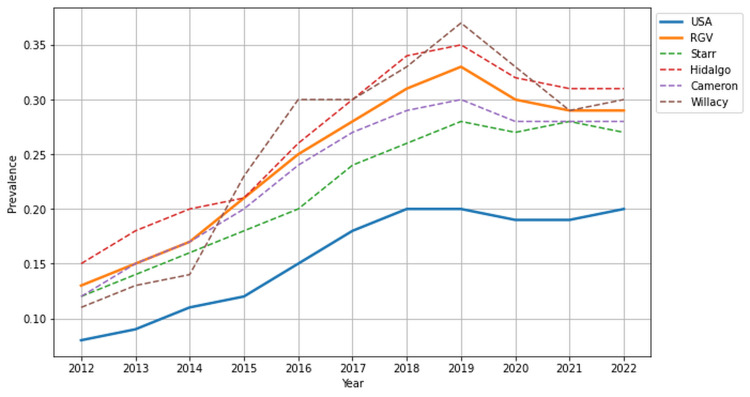
Prevalence of obesity in the USA and Rio Grande Valley (RGV) from 2012 to 2022

From 2012 to 2022, DM affected patients in the RGV (43.95%) at significantly higher rates than the national average (26.73%) (p < 0.001, 95% CI (41.27, 46.64)). Willacy County (47.76%) had the highest prevalence of DM, with Starr County following closely at 45.82%, yet all counties of the RGV surpassed the national average. Patients across the RGV struggle with obesity at higher rates than the national average (24.41% vs. 15.55%, p < 0.01, 95% CI (11.30, 37.51)). Hidalgo County (26.64%) reported the highest prevalence of obesity, followed closely by Willacy County (25.73%), Cameron County (23.45%), and Starr County (21.82%). Figures 1-2 provide a visual representation of the prevalence of DM and obesity, respectively, between the RGV, individual counties, and the national average. 

Screening

**Figure 3:**
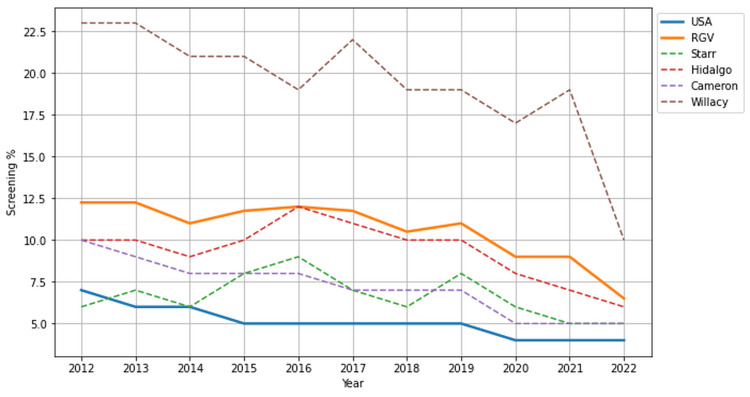
Screening rates of diabetes mellitus in the USA and Rio Grande Valley (RGV) from 2012 to 2022

Screening rates for DM in the RGV doubled the national mean, significantly outpacing the national screening rates from 2012 to 2022 (10.64% vs. 5.09%, p < 0.001, 95% CI (7.28, 13.99)). Figure 3 illustrates the screening rates of the RGV, along with its individual counties, compared to the national average. Notably, screening rates in Willacy County (19.36%) were nearly four times higher than the national average (5.09%).

Average principal cost

**Figure 4:**
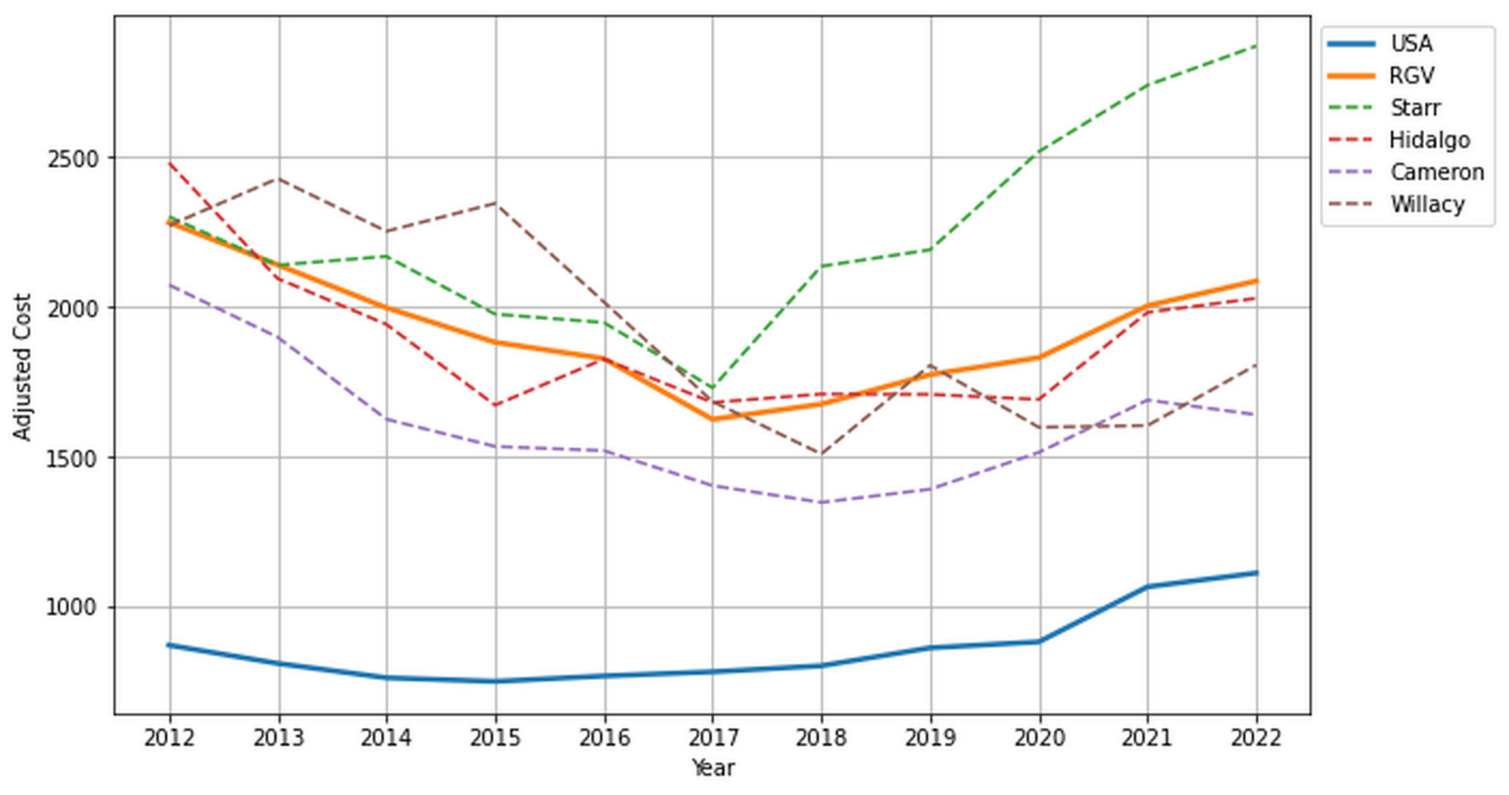
Average principal cost for diabetes mellitus treatment in the USA and Rio Grande Valley (RGV) from 2012 to 2022

The average principal cost for patients in the RGV ($1,920.45) to treat DM was significantly greater than and more than double the national average principal cost ($859.64) (p < 0.001, 95% CI (1,543.20, 2,297.71)). Among the individual counties within the RGV, Starr County demonstrated the highest average principal cost ($2,247.64), followed by Willacy County ($1,938.45), Hidalgo County ($1,892.45), and Cameron County ($1,603.27). The average principal costs shown in Figure 4 offer a comparative analysis of the RGV to its individual counties and the national average. 

Emergency department visits

**Figure 5:**
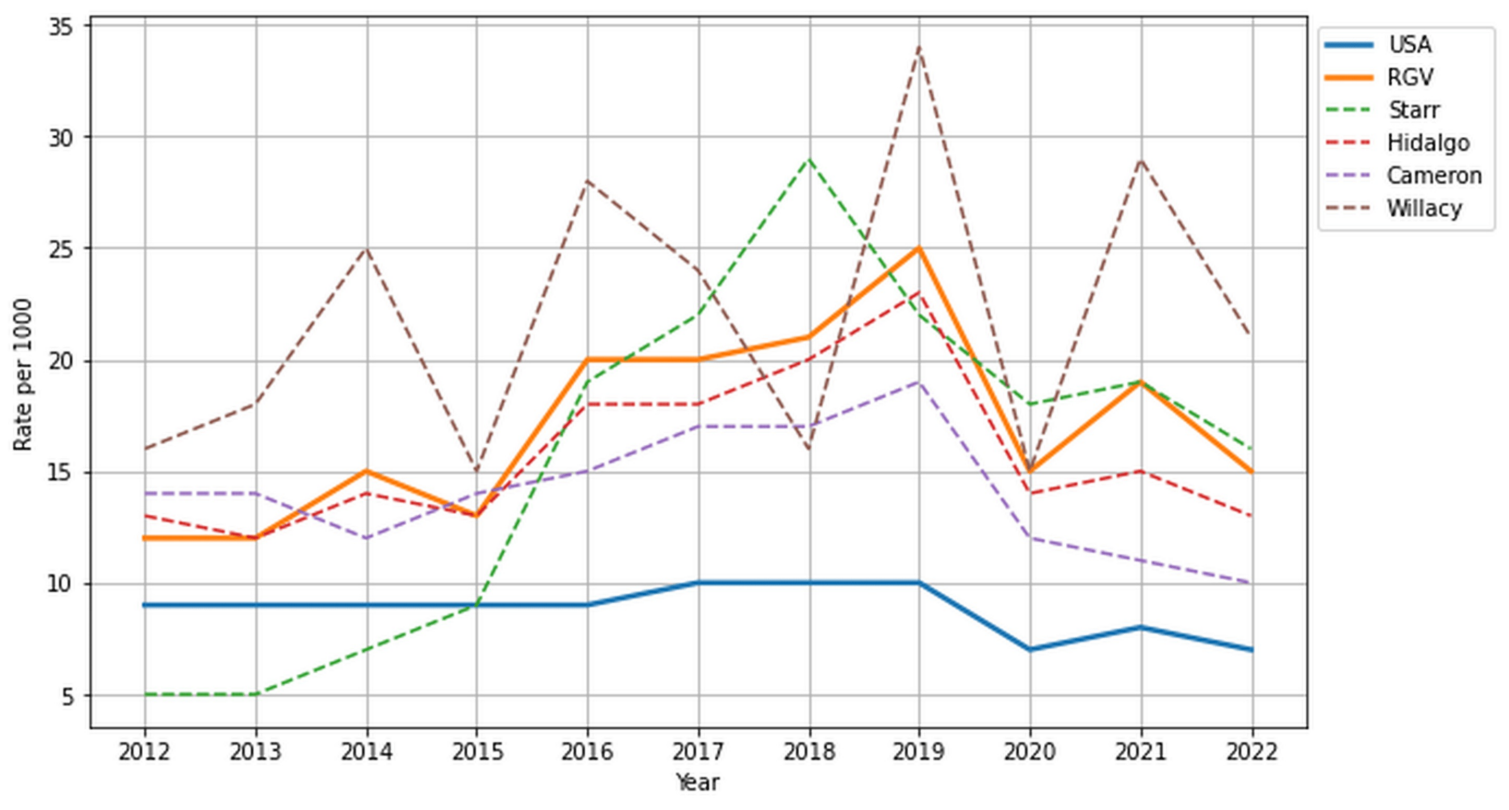
Emergency department visit rates for Centers for Medicare and Medicaid Services (CMS) beneficiaries with diabetes mellitus in the USA and Rio Grande Valley (RGV) from 2012 to 2022

In the RGV, the rate of ED visits for patients with DM averaged 16.82 per 1,000 beneficiaries from 2012 to 2022, significantly surpassing the national average of 8.82 per 1,000 beneficiaries during the same period (p < 0.001, 95% CI (9.09, 24.54)). Willacy County reported the highest rates of ED visits for patients with DM during the study period at 21.91 per 1,000 beneficiaries (Figure 5). Conversely, Cameron County exhibited the lowest ED visit rates for patients with DM at 14.09 per 1,000 beneficiaries, yet still exceeded the national rate. 

Hospitalizations

**Figure 6:**
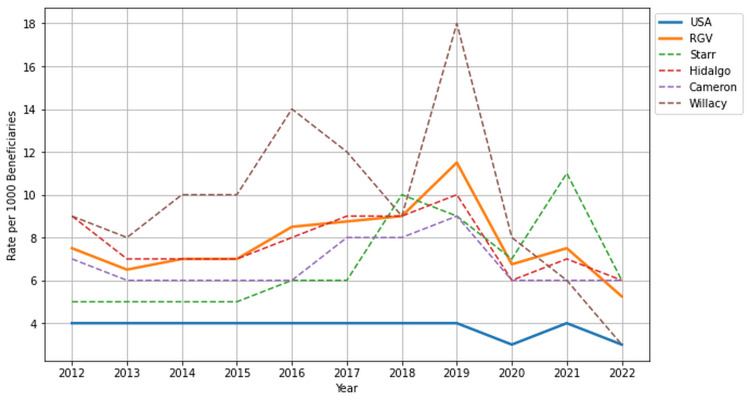
Hospitalization rate among Centers for Medicare and Medicaid Services (CMS) beneficiaries with diabetes mellitus in the USA and Rio Grande Valley (RGV) from 2012 to 2022

Patients with DM in the RGV were hospitalized at significantly higher rates (7.75 per 1,000 beneficiaries) than the national average rate of 3.82 per 1,000 beneficiaries (p < 0.001, 95% CI (4.67, 10.83)). The hospitalization rate for patients with DM in the RGV doubled the national rate due to the high rates seen in Willacy and Hidalgo counties (9.73 & 7.73 per 1,000 beneficiaries, respectively). Further analysis of the hospitalization rates of patients with DM compared across the RGV, national mean, and individual counties is depicted in Figure 6. Table 1 presents the descriptive statistics and Mann-Whitney U test for health indicators in the USA and Rio Grande Valley (RGV) regions.

**Table 1:** Descriptive statistics and Mann-Whitney U test for health indicators in the USA and Rio Grande Valley (RGV) regions Key: Statistically significant results are shown in bold (p<0.05)

Variable	County	Mean	SD	Lower CI	Upper CI	Mann Whitney U p-value
Diabetes mellitus prevalence	USA	26.73%	0.45%	25.85%	27.60%	< .001
	RGV	43.95%	1.37%	41.27%	46.64%	
	Starr	45.82%	1.53%	42.83%	48.81%	
	Hidalgo	41.91%	2.50%	37.00%	46.81%	
	Cameron	40.36%	1.23%	37.96%	42.77%	
	Willacy	47.73%	2.53%	42.78%	52.68%	
Obesity prevalence	USA	15.55%	4.50%	6.73%	24.36%	< .001
	RGV	24.41%	6.69%	11.30%	37.51%	
	Starr	21.82%	5.73%	10.58%	33.06%	
	Hidalgo	26.64%	6.66%	13.57%	39.70%	
	Cameron	23.45%	6.07%	11.57%	35.34%	
	Willacy	25.73%	8.65%	8.78%	42.67%	
Diabetes screening	USA	5.09%	0.90%	3.33%	6.85%	< .001
	RGV	10.64%	1.71%	7.28%	13.99%	
	Starr	6.64%	1.23%	4.23%	9.04%	
	Hidalgo	9.36%	1.67%	6.10%	12.63%	
	Cameron	7.18%	1.59%	4.08%	10.29%	
	Willacy	19.36%	3.47%	12.56%	26.17%	
Average principal cost	USA	$859.64	$116.25	$631.79	$1,087.49	< .001
	RGV	$1,920.45	$192.48	$1,543.20	$2,297.71	
	Starr	$2,247.64	$326.80	$1,607.12	$2,888.15	
	Hidalgo	$1,892.45	$238.12	$1,425.75	$2,359.16	
	Cameron	$1,603.27	$210.79	$1,190.13	$2,016.41	
	Willacy	$1,938.45	$321.27	$1,308.77	$2,568.14	
Emergency department visit rate	USA	8.82	1.03	6.8	10.83	< .001
	RGV	16.82	3.94	9.09	24.54	
	Starr	15.55	7.58	0.68	30.41	
	Hidalgo	15.73	3.36	9.14	22.31	
	Cameron	14.09	2.64	8.91	19.27	
	Willacy	21.91	6.24	9.67	34.15	
Hospitalization	USA	3.82	0.39	3.06	4.57	< .001
	RGV	7.75	1.57	4.67	10.83	
	Starr	6.82	2.08	2.74	10.9	
	Hidalgo	7.73	1.29	5.21	10.25	
	Cameron	6.73	1.05	4.66	8.79	
	Willacy	9.73	3.79	2.29	17.16	

## Discussion

Compared to the rest of the United States, the RGV surpasses the national average in all categories of interest in this study, including the prevalence of diabetes and obesity, screening for diabetes, the average principal cost, and diabetes-related ED visits and hospitalizations. The RGV is a unique area in the United States, with most of the residents being predominantly of Hispanic origin [7]. According to an international news service in the RGV, in 2019, the Hispanic population reached an all-time high of 91.5% [15]. Consequently, this provides an opportunity to analyze diseases and conditions disproportionately affecting this ethnic group. Additionally, the unique demographic of the RGV, predominantly Hispanic, allows the exploration of particular characteristics within the Hispanic heritage, which could be linked to conditions such as diabetes, obesity, and hypertension.

Diabetes mellitus is an ongoing struggle in the RGV, shown by the 43.95% prevalence among Medicare beneficiaries in the region from 2012 to 2022 compared to the 17.22% prevalence in the United States during the same period. According to a previous study, the prevalence of DM between 2007 and 2013 in the RGV was only 30.7%, thus signifying that DM is a worsening issue in the RGV among this specific population [16-17]. Although obesity is not the sole cause of DM, it is strongly associated with the development of type 2 DM [18]. This is particularly concerning as the prevalence of obesity is nearly 10% greater among residents of the RGV compared to the national average. According to Salinas et al., an increased concentration of Hispanic ethnicity in a population was associated with a higher prevalence of county-level obesity in communities with lower educational attainment and greater poverty [19]. This perfectly encapsulates the RGV and the social determinants of health that the medically underserved region faces. According to Logan et al., Hidalgo County is one of the most disadvantaged areas in the US, with one of the lowest average household incomes in the nation and 33.5% of residents living below the poverty line [20]. The discrepancies in the prevalence of DM and obesity in the RGV compared to the national average are likely caused by a multitude of factors, including socioeconomic status, education, language barriers, ethnic habits, and exercise frequency, among others [21]. Further research could focus on determining specific social determinants of health that contribute most to the development of DM and obesity in the RGV. Additionally, future research could investigate the most beneficial treatments for this ethnic population. 

Like DM and obesity, the screening rate for DM in the RGV (10.64%) is over double the national average (5.09%). This is presumably consequential to the elevated prevalence of DM and obesity in the RGV. Due to the increased rates in the region, it is vital that diabetes is diagnosed promptly and managed appropriately, prior to complications and hospital admissions. However, even with the higher rates of screening, the average principal cost of treating DM, ED visits, and hospitalizations for DM are significantly greater than that of the U.S. average. The average principal cost for treating DM in the RGV ($1,920.45) is more than double the national average ($859.64). This could potentially be due to the remote access to healthcare in the RGV, resulting in a lack of preventative services and appropriate treatment leading to further ED visits, hospitalizations, and expenditures for DM [16]. Creating practical and cost-effective ways to manage and treat DM can reduce the number of ED visits and subsequent hospitalizations, which would lower the cost of DM-related expenditures in the region. 

This study is not without limitations. Public Medicare beneficiary data was used for this study, which may not accurately reflect the population of the RGV. Secondly, the ‘Mapping Medicare Disparities by Population’ tool does not differentiate between type 1 DM and type 2 DM [12]. However, since most Medicare beneficiaries are older adults and type 2 DM is predominant in this age group, the data remain highly relevant to the objectives of our study. Thirdly, the results could potentially underestimate the prevalence of DM in the RGV considering the significant portion of residents lacking legal status, health insurance, and financial means to acquire adequate screening and treatment [22]. Future research should study hospital-acquired data in the RGV to enhance the generalizability of these findings, furthering the understanding of the landscape of DM in the region.

## Conclusions

In conclusion, this study highlights DM as a significant public health concern in the RGV, an economically disadvantaged and medically underserved area of the U.S. This study also analyzes several metrics associated with DM and illustrates a comprehensive view of the overall health environment in the RGV, including higher rates of obesity, increased screening rates for DM, increased ED visits and hospitalizations of patients with DM, and an increased average principal cost to treat DM in the RGV when compared to the national average. The elevated prevalence of DM within the RGV underscores the need for comprehensive strategies that target prevention, early detection, and effective management of DM and its associated complications. Implementing targeted interventions aimed at mitigating the effects of DM is crucial for improving the overall health and well-being of the residents of the RGV.

## References

[1] American Diabetes Association Professional Practice Committee (2024). 2. Diagnosis and classification of diabetes: standards of care in diabetes-2024. Diabetes Care.

[2] Glovaci D, Fan W, Wong ND (2019). Epidemiology of diabetes mellitus and cardiovascular disease. Curr Cardiol Rep.

[3] Balakumar P, Maung-U K, Jagadeesh G (2016). Prevalence and prevention of cardiovascular disease and diabetes mellitus. Pharmacol Res.

[4] Hassan S, Gujral UP, Quarells RC (2023). Disparities in diabetes prevalence and management by race and ethnicity in the USA: defining a path forward. Lancet Diabetes Endocrinol.

[5] Wild S, Roglic G, Green A, Sicree R, King H (2004). Global prevalence of diabetes: estimates for the year 2000 and projections for 2030. Diabetes Care.

[6] Chen Y, Zhou X, Bullard KM, Zhang P, Imperatore G, Rolka DB (2023). Income-related inequalities in diagnosed diabetes prevalence among US adults, 2001-2018. PLoS One.

[7] Salcedo MP, Gowen R, Rodriguez AM (2023). Addressing high cervical cancer rates in the Rio Grande Valley along the Texas-Mexico border: a community-based initiative focused on education, patient navigation, and medical provider training/telementoring. Perspect Public Health.

[8] Innis-Whitehouse W, Wang X, Restrepo N, Salas C, Moreno K, Restrepo A, Keniry M (2018). Kaposi sarcoma incidence in females is nearly four-fold higher in the Lower Rio Grande Valley compared to the Texas average. Cancer Treat Res Commun.

[9] Bowden VM, Wood FB, Warner DG, Olney CA, Olivier ER, Siegel ER (2006). Health information Hispanic outreach in the Texas Lower Rio Grande Valley. J Med Libr Assoc.

[10] Bialaszewski RP, Gaddis JM, Martin B, Dentino P, Ronnau J (2023). Bridging bone health: osteoporosis disparities in the Rio Grande Valley. Cureus.

[11] Garza N, Uscamayta-Ayvar M, Maestre GE (2020). Addressing neurocognitive disorders, dementias, and Alzheimer’s disease in colonias of the lower Rio Grande Valley: establishing a research foundation using promotores. Ethn Dis.

[12] Centers for Medicare & Medicaid Services Office of Minority Health (CMS OMH) (2024). The Mapping Medicare Disparities Tool, Technical Documentation [Internet]. CMS. The Mapping Medicare Disparities Tool, Technical Documentation.

[13] Vergil NS (1978). The International Classification of Diseases: Ninth Revision (ICD-9). Ann Intern Med.

[14] World Health Organization (2004). ICD-10: International Statistical Classification of Diseases and Related Health Problems: Tenth Revision, 2nd Ed.

[15] Reyes D (2022). New research shows economic power of Hispanic households in RGV. https://riograndeguardian.com/new-research-shows-economic-power-of-hispanic-households-in-rgv/.

[16] De La Garza R, Rodrigo H, Fernandez F, Roy U (2019). The increase of HIV-1 infection, neurocognitive impairment, and type 2 diabetes in the Rio Grande Valley. Curr HIV Res.

[17] Millard AV, Graham MA, Mier N (2017). Diabetes screening and prevention in a high-risk, medically isolated border community. Front Public Health.

[18] Leong KS, Jayasinghe TN, Wilson BC (2020). High prevalence of undiagnosed comorbidities among adolescents with obesity. Sci Rep.

[19] Salinas JJ, Rocha E, Abdelbary BE, Gay J, Sexton K (2012). Impact of Hispanic ethnic concentration and socioeconomic status on obesity prevalence in Texas counties. Int J Environ Res Public Health.

[20] Logan RI, Castañeda H (2020). Addressing health disparities in the rural United States: advocacy as caregiving among community health workers and promotores de Salud. Int J Environ Res Public Health.

[21] Zhang K, Reininger B, Lee M, Xiao Q, Bauer C (2020). Individual and community social determinants of health associated with diabetes management in a Mexican American population. Front Public Health.

[22] Kuruvilla R, Raghavan R (2014). Health care for undocumented immigrants in Texas: past, present, and future. Tex Med.

